# An Introduction to the Inverted/Flipped Classroom Model in Education and Advanced Training in Medicine and in the Healthcare Professions

**DOI:** 10.3205/zma001045

**Published:** 2016-05-17

**Authors:** Daniel Tolks, Christine Schäfer, Tobias Raupach, Leona Kruse, Antonio Sarikas, Susanne Gerhardt-Szép, Gertrud Kllauer, Martin Lemos, Martin R. Fischer, Barbara Eichner, Kai Sostmann, Inga Hege

**Affiliations:** 1 Klinikum der Universität München, Institut für Didaktik und Ausbildungsforschung in der Medizin, München, Deutschland; 2 Philipps Universität Marburg, Fachbereich Medizin - Studiendekanat, Marburg, Deutschland; 3 Universitätsmedizin Göttingen, Studiendekanat, Medizindidaktik und Ausbildungsforschung, Göttinge, Deutschland; 4 University College London, Health Behaviour Research Centre, London, UK; 5 CAU Kiel, Medizinische Fakultät, Studiendekanat, Koordination E-Learning, Kiel, Deutschland; 6 Technische Universität München (TUM), Fakultät für Medizin, Institut für Pharmakologie und Toxikologie, München, Deutschland; 7 Goethe-Universität, Carolinum Zahnärztliches Universitäts-Institut gGmbH, Poliklinik Zahnerhaltungskunde, Frankfurt am Main, Deutschland; 8 Goethe-Universität Frankfurt FB 16 Medizin, Dr. Senckenbergische Anatomie- Anatomisches Institut II, Frankfurt, Deutschland; 9 RWTH Aachen, Metizinische Fakultät, Audiovisionelles Mediencentrum, Aachen, Deutschland; 10 Universität Ulm, Medizinische Fakultät, Studiendekanat Molekulare Medizin, Kompetenzzentrum eLearning in der Medizin BW, Ulm, Deutschland; 11 Charité - Universitätsmedizin Berlin, Dieter Scheffner Fachzentrum für medizinische Hochschullehre und evidenzbasierte Ausbildungsforschung, Berlin, Deutschland; 12 Geisel School of Medicine at Dartmouth, USA

**Keywords:** inverted classroom, flipped classroom, medical education, educational video, Open Educational Resources, MOOCs, blended learning, screencasts, podcasts, E-Learning

## Abstract

In describing the inverted classroom model (ICM), the following paper is meant to provide an introduction to the subject matter and to serve as a practical guide for those wishing to employ its methods in basic and advanced medical training and education. The ICM is a blended-learning method in which a self-directed learning phase (individual phase) precedes the classroom-instruction phase. During the online phase, factual knowledge is imparted that serves as a basis for the classroom phase. The classroom phase should subsequently be used to assimilate and implement the previously gained knowledge. In contrast, traditional course concepts impart factual knowledge in lectures, for example, or in other face-to-face teaching formats and are followed by the students’ self-instruction in order to assimilate this knowledge. The goal of the ICM is the shift from passive learning to accelerated learning in order to foster learning at cognitively demanding levels such as analysis, synthesis and evaluation.

The concurrent increase in production and use of screencasts and educational videos, the Open Educational Resources “movement” and the widespread use of Massive Open Online Courses (MOOCS) have contributed to the increased dissemination of the inverted-classroom method. The intention of the present paper is to provide an introduction to the subject matter and simultaneously to offer a short overview of important projects and research results in the field of medical education and other health professions. Furthermore, an outline is given of the advantages and disadvantages of the model as well as its potential benefit to the future of medical education and training.

## Zusammenfassung

Dieser Artikel beschreibt die Inverted-Classroom-Methode (ICM) im Sinne einer Einführung in die Thematik und soll als Praxisleitleitfaden für diejenigen dienen, die diese Methode in der medizinischen Aus-, Fort- und Weiterbildung einsetzen möchten. Es handelt sich bei der ICM um einen Blended-Learning-Methode, bei dem eine Selbstlernphase (individuelle Phase) vor die Präsenzunterrichtsphase gesetzt wird. In der Online-Phase wird Faktenwissen vermittelt, das als Grundlage für die Präsenzphase dient. Die Präsenzphase soll anschließend dafür genutzt werden, das erlernte Wissen zu vertiefen und anzuwenden. Dem gegenüber stehen die traditionellen Kurskonzepte, in denen das Faktenwissen beispielsweise in Vorlesungen oder in anderen Präsenzunterricht-Formaten vermittelt wird und die Vertiefung dieses Wissens durch die Studierenden im Anschluss daran im Selbststudium stattfinden soll.

Das Ziel der ICM ist die Verschiebung des passiven Lernens hin zum aktivierenden Lernen, um das Lernen auf kognitiv anspruchvollen Ebenen wie Analyse, Synthese und Evaluation zu unterstützen.

Dabei haben die gestiegene Produktion und Nutzung von Screencasts und Lernvideos, die „Bewegung“ der „Open Educational Resources“ und die verbreitete Nutzung von „Massive Open Online Courses“ (MOOCs) zu einer gestiegenen Verbreitung der Inverted-Classroom-Methode beigetragen. Der Artikel soll als Einführung in die Thematik dienen und dabei eine kurze Übersicht über wichtige Projekte und Forschungsergebnisse in der medizinischen Ausbildung und in weiteren Gesundheitsberufen geben. Außerdem werden die Vor- und Nachteile der Methode und ihr potentieller Nutzen für die zukünftige medizinische Aus- und Weiterbildung dargestellt.

## 1. Introduction

In light of increasing division of labour in healthcare, the training and acquisition of both profession-specific and interprofessional competencies have been attributed growing significance, creating the need to test and establish specific teaching formats [1]. Despite ever more complex and interconnected healthcare systems, an increase in patients’ active self-responsibility and innumerable pedagogical and technological innovations, educational systems have not reacted adequately to these new demands. Many authors, not lease the German Council of Science and Humanities, have therefore urged a rethinking of traditional medical education [2], [3], [4], [5], [6], [7], [8], [9]. Student-centred learning activities, such as problem-based and research-based learning, are becoming increasingly significant in view of the numbers of students achieving unsatisfactory levels of competence in critical thinking, communication and writing abilities and complex clinical decision making, for example [10]. The Council of Science and Humanities arrived at a positive evaluation of the various model and reformed courses of study attempting to effectuate a comprehensive reorganisation of medical studies in content and structure as well as methods and didactics [1]. 

The persistent pervasiveness of instructor-centred learning formats is not only to be found in medical education but in all of the health professions [10], [11], [12].

Although alternative teaching and instruction formats have already been designed and their effectiveness deemed positive in empirical evaluation, the lecture remains the most practised means of transmitting knowledge [7], [8]. In its essence, however, learning is not a question of transmitting information but, moreover, a question of processing information [7]. In traditional instruction units, referred to as “chalk and talk classes” by Becker and Watts, the teaching party presents material in the form of a lecture [13]. As appropriate, questions may be permitted or short processing periods for the students may be integrated into the lecture. The knowledge-assimilating and most essential analysis of the lecture’s contents takes place in the subsequent self-instruction phase, in which the student works alone on concrete tasks. It is during the transfer of knowledge conveyed in the lectures, however, that most questions arise [14]. Of further disadvantage in the traditional lecture is the low level of motivation among students to attend lectures as well as their often heterogeneous knowledge [15]. The Inverted Classroom Model seems to be an eligible instrument for greater facilitation of student-centred and interprofessional learning [3], [4], [5], [6], [7].

## 2. The inverted classroom concept

Since the end of the 1980s, the expectations for the application of multimedia in basic, advanced and further education and training have peaked in repeating phases of enthusiasm, only to decline again thereafter. Known for some time now, the flipped or inverted classroom model seems to be contributing to a new rise in expectations in the context of a further “hype cycle for technology” [16], [http://www.gartner.com/newsroom/id/2819918 cited 12. Januar 2015]. The term “flipped classroom” describes the use of the model’s methods in primary and secondary education [17], while “inverted classroom” is used to denote the model’s application in higher education [18], [19]. Despite the greater attention it has been receiving of late, it is not a new concept, having been ranked by the Horizon Report 2014, for example, as one of the most significant teaching and learning technologies developed in further education [20].

**Figure 1:**
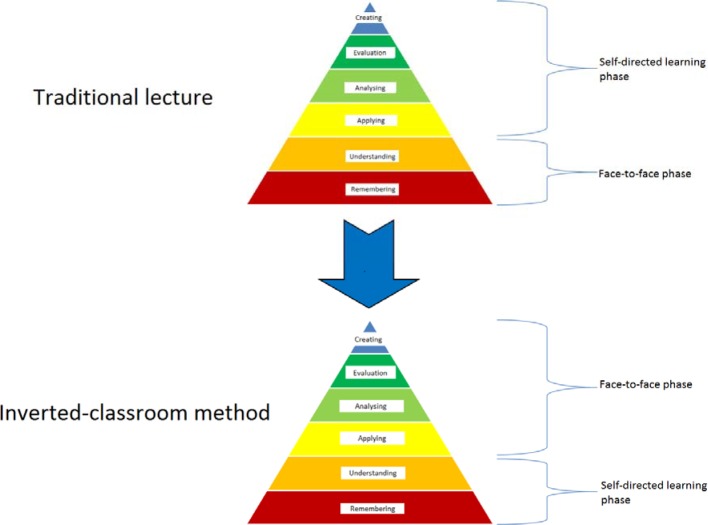
The traditional lecture and the inverted classroom model in schematic comparison according to Bloom’s revised taxonomy (31)

The Inverted Classroom Model (ICM) involves a blended-learning method in which a self-directed learning phase (individual phase) takes place before the classroom-learning phase (see Figure 1 (Fig. 1)).

**Figure 2:**
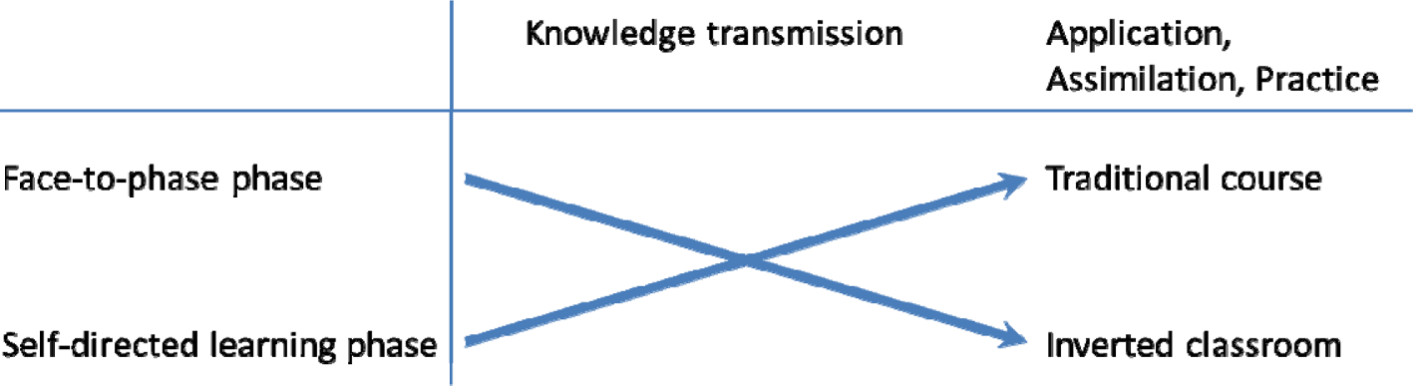
Reversal of learning phases in the flipped classroom method (15)

In the online phase, factual knowledge is conveyed that serves as a basis for the classroom or face-to-face phase. The subsequent classroom phase should then be used to assimilate and implement the acquired knowledge. The traditional course concept foresees the gathering of factual knowledge in lectures or other face-to-face formats of conveyance after which course participants deepen and, on occasion, implement said knowledge on their own. The “Inverted Classroom Model” switches the allocation of the respective tasks of the two phases (see Figure 2 (Fig. 2)). 

## 3. Theoretical basis

The goal of the Inverted Classroom Model is a shift from passive learning to accelerated learning in the classroom phase in order to accelerate the acquisition of more demanding competencies such as analysis, synthesis and evaluation. In the terms of Bloom’s revised taxonomy [21], this means that students accomplish lower-order cognitive processes (acquisition of knowledge and comprehension) independently prior to classroom instruction in order to subsequently execute higher cognitive learning process (use of knowledge, analysis, synthesis and evaluation) in the classroom phase, during which they can be directly supported by peers and instructors. Figure 1 (Fig. 1) depicts this inversion of learning domains. 

Lage, Platt and Treglia (19) assumed that established teaching formats were not compatible with the different learning styles of students. 

A meta-analysis by the U.S. Department of Education confirmed that the use of blended learning (i.e., the combination of the online and self-directed learning phase with classroom learning) leads to better results than exclusively online seminars or exclusively classroom teaching [22]. A further study showed that students prefer collaborative or interactive learning together with other students or with the lecturer to individual self-directed learning phases [23]. One important finding is that online learning material shows the greatest effect in addressing lower-order cognitive skills, such as learning and comprehension [24]. With regard to online lectures, Nast and colleagues and Burnette and colleagues showed that there was no difference between online lectures and face-to-face lectures where the students’ knowledge retention is concerned [25], [26]. 

The defining aspect of the Inverted Classroom Model is the promotion of accelerated learning. Through the self-directed-learning phase and enhancement in discussion during the classroom phase, accelerated learning aspects such as teamwork, debate and self-reflection are fostered. Accelerated learning increases learning success, motivation and positive attitudes and facilitates higher-order cognitive learning processes, problem-solving competence and the critical analysis of learning content [27], [28], [29], [30].

## 4. Developmental trajectories

The concept of the flipped-class method was first mentioned in 1998 in the book “Effective Grading” by Barbara Walvoord and Virginia Johnson Anderson. Their idea was to make select basics from the disciplines history, physics and biology available to students online prior to the actual classroom instruction and to expand and anchor the knowledge gained in the classroom setting [31]. This would re-allocate the transfer of facts to the online phase in order to shift the focus onto the application of this newly gathered knowledge to the classroom phase. To assure the appropriate preparation of the students, they were given assessment tasks prior to classroom instruction. 

Almost simultaneously, Maureen Lage, Glenn Platt and Michael Treglia tested the inverted classroom method in higher education in an introductory economics course [18]. Assuming that traditional instruction formats were incompatible with modern forms of learning, they asked students to prepare for the classroom phase by watching video recordings in which economic principles were conveyed using case studies and simulation games. The authors observed a rise in motivation and satisfaction with the course concept and an increase in interaction and participation in verbal contributions (particularly among female participants). 

To date, the Flipped or Inverted Classroom Model has been applied chiefly in primary, secondary and higher education. Concepts for adult education have been rare thus far. According to Handke and Schäfer, the ICM has spread across various disciplines, particularly in the United States [32]. Some faculties have reorganised curricula entirely to the Inverted Classroom Model [33], [34], [35]. There has also been a noticeable increase in the curricular integration of the ICM, in mathematics for example [36]. In this context, Treeck points out that the ICM is often applied but not designated as such [14]. Conversely, it would seem that there are many concepts in the meantime that are designated as ICM but do not correspond to the model’s methods. 

Three developments have significantly contributed to the increased spread of the Inverted Classroom Model:

- the rise in production and use of screencasts and instructional videos
- the “Open Educational Resources” movement
- Massive Open Online Courses (MOOCs)

These developments are outlined in the following pages.

### 4.1. Screencasts and educational videos

Acceptance and use of online videos have risen sharply in recent years. 25 percent of the world’s population views online video content daily on their PCs, laptops, tablets or smart phones. In Germany, users primarily stream videos. With a weekly use of 79 percent, 14- to 29-year-olds prove to be particularly fond of video. This is due in part to new technological developments making the production and distribution of self-made videos easier but also to a new understanding of education that was expedited especially by the Khan Academy, the open educational resources movement and by the development of MOOCs. 

The Khan Academy, with its new style of educational video, has strongly influenced and advanced inverted-classroom and MOOC activity. In the so-called Khan instructional videos, the focus was moved from the traditional, elaborate high-level production to simpler educational video productions. The lecturer often does not even appear on screen, and the lecture recordings (sound and/or picture), PowerPoint slides, recordings of blackboards, whiteboards, etc. are employed to convey content in the simplest way. 

One of the more popular media in this segment is the so-called screencast, in which a software programme records the content of the monitor and the comments made by the lecturer in real-time. In this first step, the videos convey factual knowledge to the viewer. Afterwards, this knowledge is applied in tasks and quizzes, through the writing of reports and through participation in forum discussions. 

#### 4.2. Open Educational Resources

In comparison with the worldwide online community, open educational resources in the education sector receive less attention in German-speaking Europe. The underlying conviction of the OER movement is that learning material should be freely accessible to the general public.

In accordance with this tenet, educational videos and material are provided without any commercial interests. The number of freely available or “open” lecture videos on the internet has risen notably in recent years. The users of this teaching and learning material are encouraged by the authors to integrate the media into their curricula. Such developments make it possible for German academic institutions to work using filmed lectures from Harvard University, for example. Most authors and users have adopted the philosophy that it is more sensible and efficient to transmit an already existing lecture by experts and colleagues via video than to create the material themselves anew. The more open-access content is created, the greater the chance that the focus will shift from the transmission of information to the processing of information [7]. 

#### 4.3. MOOCs

 MOOC (massive open online course) designates a course that takes place online, usually addressing a subject over the space of weeks and in which, principally, anyone may participate. MOOCs generally have several organisers or moderators and are coordinated through a common website or learning management system. In part, the actual content of the event is decided in cooperation with participants through their contributions and discussions in forums, chats, social networks, video conferences and occasionally in local face-to-face meetings. The amount of different types of MOOCs (cMOOCs or xMOOCs) is constantly rising and their use has become quite widespread in Europe in the meantime [http://openeducationeuropa.eu/en/european_scoreboard_moocs cited 12. Januar 2015]. The MOOC movement also profits from open educational resources and the new type of instructional video developed by the Khan Academy. Most MOOC concepts are based on the transmission of knowledge through open-access educational clips, screencasts and filmed lectures. Additionally, the capability of making videos quickly and cost-efficiently has expedited the widespread use of educational videos in MOOCs considerably. The inverted classroom model can be used in combination with MOOCS (MOOC wrapping) [37]. MOOC content conveyed through educational videos could be used in the self-directed learning phase. The facts accumulated there can then be further assimilated in the classroom phase. 

## 5. Implementation of the Inverted Classroom Model

In the online phase, learning material is made available to course participants, often encompassing short educational videos. The use of educational videos is, however, not absolutely necessary. If the decision is made to implement educational videos, the instructors may choose to create their own short instructional clips or to draw on existing videos, should they be openly accessible. Screencasts or short lecture recordings are normally used in creating in-house educational clips; other forms of content display are also possible online (e.g., scripts, books, text excerpts). There are varying opinions as to the optimal length of the clips. Khan deems the optimal video length to be between six and ten minutes, whereas Lindner suggests that videos should be kept to shorter lengths when incorporating many visual elements, as Kerres has emphasised as well [38], [39], [https://www.youtube.com/watch?v=Ohu-5sVux28&feature=youtube_gdata_player cited 13. Januar 2015]. Handke & Franke speak of e-lectures conceived as “maximally 20-minute educational videos” and whose “content is closely tied to the virtual sessions but also offers additional information” [40]. This length corresponds to the maximal attention span of the average learner and should therefore be seen as length limit [41]. A large-scale survey found the best attention span among users of MOOC videos to be six minutes [42]. It is, however, difficult to keep to this very short time span, particularly when dealing with complex subject matter. 

In the use of learning content for online instruction, there is a noticeable trend toward easily created instructional clips. In comparison to the elaborate production of high-quality educational video material, the simpler version is more time- and cost-efficient, flexible and facilitates quick updates. In the classroom phase, content delivery can be flexibly enhanced and supplemented by filming individual sessions of the classroom phase and can then also be used in other courses. 

Video use can be made a central aspect of the ICM. As previously described, it is equally possible to implement other learning material. In other words, the ICM is not “video learning” [19], [36]. 

In the context of local curricular focus areas, many instructors will want to create the specific preparation material for their face-to-face sessions themselves. As far as generic material that is taught independent of local practise in similar intensity is concerned, however, it is possible to make use of open-access external sources. An overview of this type of freely available learning material, which may be used for instruction in compliance with the respective licensing requirements, is offered in the following description. Although the authors of the material encourage its use, they strongly suggest that a case-by-case review be made with regard to quality, didactic value, appropriateness for the planned instructional event, as well as utilisation rights principles.

### Online lectures & open educational video sites

Some of the following providers allow the integration of their videos into other websites. Videos can be linked or integrated directly into websites or learning management systems.

- **The Khan Academy: **With the support of the Gates Foundation, Sal Kahn’s organisation is currently the leading provider of free tutorials on various topics and at various levels of difficulty.
- **The OpenCourseWare Consortium: **This is also an extensive database with many open-access video lectures.
- **Academic Earth:** This website offers hundreds of free videos from leading universities such as Yale, Stanford, Harvard, etc.
- **TED – Ideas worth spreading: **A collection of lecture contributions with an emphasis on “Technology, Education and Design” from this non-profit organisation.

Further providers of open educational resources:

- **OER Commons: **The OER Commons is a structured database offering a collection of approx. 30,000 learning and instructional resources from other websites.
- **The DiscoverEd Search Engine Creative Commons: **A search engine designed solely for the purpose of searching for Creative Commons/OER materials.
- **The OER Dynamic Search Engine page Wikispaces: **Wikispaces.com , already with a great number of existing wikispaces, is a web-hosting site where education experts create their own web presence.

The classroom phase should serve as a forum for application of previously delivered factual knowledge. This face-to-face phase leans more strongly toward tasks, interaction and questions. The instructor should not repeat content from the online phase during the classroom phase. Course participants must fully understand that preparation for the classroom phase and assuming responsibility for it represents a central part of the concept [14]. In the classroom phases, group methods such as pair work, group discussion, problem-based learning, think-pair-share, buzz groups or snowballing can be used. 

 Playful approaches to higher education are being increasingly discussed, as yet, primarily in the context of audience response systems and gamification. The face-to-face session in the inverted classroom can be used for lecture hall games in which students test their knowledge in playful quiz situations and groups compete with each other, for example [43]. 

## 6. Advantages

The general advantage of the Inverted Classroom Model as opposed to the classic lecture lies in removing the transmission of purely factual knowledge from the classroom phase, thus making more time available for the application of knowledge and for transfer accomplishment in this face-to-face phase, including large lecture events. This gives teachers and learners, with their individually constructed knowledge, the possibility to exchange and reflect on their procedures and experiences, even within a large gathering, and to co-create the instructional event accordingly. In publications on the subject, the greater involvement of students is seen as a fundamental advantage of the ICM [35].

According to the University of Southern California, the implementation of the Inverted Classroom Model offers the following additional advantages [https://cst.usc.edu/teach/strategies/the-inverted-classroom/ cited 13. Januar 2015]:

- the possibility for students to appropriate content at their own learning pace;
- the possibility of self-assessment for students and lecturers, through the introduction of small tasks and quizzes into the process;
- the possibility to receive direct feedback;
- the possibility to interact with the lecturer;
- the possibility to work on, answer and discuss questions that arise.

## 7. Disadvantages

The disadvantages of the ICM emerge chiefly when the requirements for the successful implementation of the methods are not fulfilled. Therefore, this section will deal with some of the conditions upon which, in the view of the authors, the success of the ICM depends.

Firstly, it is evident that the effective implementation of the ICM requires that students prepare themselves with the aid of learning materials made available to them previously. Some reasons for inadequate acquisition of knowledge in the online or self-directed learning phase may be lack of time, lack of motivation, or highly complex content [36]. In this case, lecturers would need to depart from the ICM in favour of traditional knowledge transmission in the classroom phase. It follows that course participants will not have sufficient space and time to assimilate and apply the factual knowledge that was actually presupposed. The problematic nature of this lack of assimilation then also comes to light when the classroom phase does not build on the online phase. Consequently, assimilation does not take place and the course participants’ motivation to prepare for the following course declines. The significance of a careful selection of preparatory material is correspondingly great. The use of the aforementioned open resources saves time. Simultaneously, there is a danger that the online material and classroom instruction will not be congruent. Instructors must 

know the content of the online resources they have selected and identify with it in order for a classroom-phase discussion of the content’s validity to arise. Additionally, the resources for the preparation phase should be in harmony with local curricular needs and the National Competency-Based Catalogue of Learning Objectives for Medical/Dental Education. A further requirement for the ICM is that instructors must initially expend more effort (selection of teaching material; content- and format-related planning of the classroom phase). This said, the already created contents can be used for several courses, and the effort in total can be reduced through the utilisation of existing instructional material. A further requirement for the effective implementation of the model is an initial instruction of the students, in which the aforementioned conditions are explained and operationalised in practical examples. 

## 8. Research results

Research results on some aspects of the ICM do already exist. The following studies are only a small excerpt and refer to the overview by Bishop and Verleger [44] for the most part. They in make no claims to being complete. It is of particular note that the researched contexts are very diverse: Publications on the implementation of the ICM in primary/secondary education, in higher education, in relation to medical and non-medical content, as well as research from various countries is available. The results can potentially not all be generalised for all contexts. For example, it has yet to be definitively shown that preparation for the classroom phase without specific preparatory tasks will lead to successful learning at all. 

Many of the works document the use of filmed lectures in teaching, focussing on material implementation and on participation in face-to-face instruction. Fischer and Spannagel have summarised several studies on the altogether positive reception of the Inverted Classroom Model. In this context, Loviscach points out that the IC Model is particularly suitable in identifying knowledge gaps and problems that were left undetected in conventional lectures [36]. A prospective study by Raupach and colleagues showed that the use of podcasts in combination with quizzes prior to medical instruction has a positive influence on short-and medium-term knowledge retention [45]. 

Lage and colleagues have attested to the positive reaction to the new course concept among students, who report that they enjoy learning more and actually use the content from the online phase during the classroom phase. Furthermore, the students feel less inhibited to actively take part in discussions. They generally display higher activity levels in the classroom phase in comparison to traditional lecture situations. Lecturers have observed a more active participation among students in discussions. Most students report preferring the ICM to traditional instruction. In a study by Deslauriers and colleagues, it was shown that the use of the flipped classroom method can lead to significant learning improvements among physics pupils [46].

## 9. The inverted classroom in medical education and health professions

As some studies on learning results and medical students’ participation have indicated, there are several deficits in traditional higher education.

It is, for example, well known that students’ attention decreases after only ten minutes and after an average of 15 to 20 minutes is almost fully lost. Directly following a lecture, students only remember approximately 20% of the transmitted content [47]. In light of these findings alone, there is an urgent need for a teaching reform that provides for more effective knowledge transmission than in traditional lectures. In the case of the education and training of physicians, the necessity of practising clinical decision making is an additional aspect. This can be done very effectively by means of casework with online-communicated cases, having the further inherent advantage that the students can learn from their mistakes without endangering patients [48]. 

The Inverted Classroom Model has already been implemented in several projects in medical education as well as other health professions. The following is a presentation of some of these projects, making no claim to being complete. The projects presented are those that have been mentioned and cited most frequently.

In medical education, there have been a few studies in the area of the ICM. Using the Inverted Classroom Model for gynaecological oncology, Morgan and colleagues arrived at good results with regard to student acceptance as well as with reduction of knowledge transmission duration [49]. At the Northwestern University Feinberg School of Medicine in Chicago, the Inverted Classroom Model was implemented in case-presentation training, providing all preparatory, theoretical content in an online phase. The actual presentation was trained in the classroom phase under instructor supervision. As a result, the students’ examination performance improved significantly compared with that of the previous semester’s [50].

Further studies were also able to show an improved learning effect in the physiology faculty, with mixed levels of acceptance among students however [51], [52]. The interpretation of these results is limited due to shortcomings stemming from the respective study designs (mostly historical controls; lacking or, at best, random congruence between instructional material and exam content). Several studies and projects featuring ICM implementation in other health professions exist as well. 

In Germany, the Inverted Classroom Model was applied at the Ludwig Maximilian University of Munich (LMU) in the scope of academic instruction qualification, offering further good results in student acceptance [53]. Albeit with few study participants (n=40), the Inverted Classroom Model also proved appropriate for this segment of education and advanced training. In addition to this, the ICM is being tested in LMU’s faculty of general medicine. 

The school of dentistry at the Goethe University of Frankfurt implemented the ICM in conservative dentistry in the form of the “P@L” project, which was accepted as an example of good practice by the German Rector’s Conference. One particularity of the ICM used here is that learning takes place in small groups in problem-based-learning scenarios (PBL). Gerhardt-Szép was able to show that particularly collaborative and self-directed learning in this context received positive evaluations from the students [54].

At their annual conference in 2014 and 2015, the Association for Medical Education in Europe (AMEE) presented the AMEE Initiative: Research Papers: “Flipped-Classroom – Technology and Assessment for Learning”, in response to the advantages offered by the new teaching and learning method. Participants were able to acquire relevant information prior to the conference in an online phase, allowing them to engage in more intense discussion with speakers at the event itself. The event, however, only met with patchy success: According to their own statement, only one symposium guest had viewed the online material beforehand. 

The communication of similar competencies is also necessary in other health professions. Critical thinking and teamwork are also important factors for nursing [53], [54], [55], [56], [57].

At the University of Bradford, these competencies in particular are conveyed using the Inverted Classroom Model. Results here suggest that the use of the ICM not only leads to improved learning performance but also facilitates problem-solving competency and (interdisciplinary) teamwork. According to the authors, the inverted classroom model presents an appropriate form of instruction because it targets complex-problem solving and the facilitation of problem-solving competencies and teamwork [58]. 

One study within the field of physiotherapy also attested to an improvement in learning performance, albeit with low-level acceptance of the learning format among students [59]. 

 At the University of North Carolina School of Pharmacy, the traditional teaching concept was changed to the Inverted Classroom Model. Three elements explicitly were implemented: facts were transmitted online, the focus of teaching and learning methods were on student-centred communication and assessment formats were used. For the online phase, filmed lectures averaging 34 minutes in length were prepared, with the intention of delivering the most important content in compressed form, and literature was included as a supplement. In the classroom phase, learning activities such as feedback and Q&A, microlectures, “clicker” systems (audience response), pair-and-share, presentations, discussions and quizzes were used to facilitate assimilation of the knowledge gathered by students in the self-directed learning phase, to foster critical thinking and to stimulate discussion. The students (n=150) showed significantly improved exam results in comparison with the previous, traditionally instructed semester. Furthermore, greater course concept satisfaction (93.1%) and higher attendance rates were observed [4]. 

Prober and Heath implemented the Inverted Classroom Model in a biochemistry curriculum and also showed significantly improved learning results in comparison with the previous semester. They too observed very high satisfaction levels and a 30% to 80% higher attendance rate [5]. Good results were also recorded in the framework of pharmacy studies at Shenandoah University in Winchester, Virginia in a renal pharmacotherapy module. In addition to improved test results in comparison with the previous year, the use of the ICM produced high satisfaction levels (80%) among students [60]. A further study within pharmacokinetics also delivered good results in learning performance [61]. 

## 10. Discussion and outlook

A departure from traditional lecture formats toward learner-centred instruction appears to be advisable in medical education and in the education and training of other health professions. This demands the shift of fact transmission to an online, self-directed learning phase and of the application of knowledge and exercises to the classroom phase. In view of the outlined parameters and published research data, future instruction should not limit itself to the transmission of purely of factual knowledge, but should provide the space to use this knowledge for problem solving in practice. Van der Vleuten and Driessen argue for a shift of focus from information transmission to information processing and emphasise that this can be facilitated using the ICM [7]. According to the authors, the ICM has the potential to bolster the imparting of competencies such as clinical reasoning, critical thinking, communication behaviour and the capacity for teamwork. All of these outcomes are of great significance in patient-centred healthcare. Further studies are necessary, however, in order to confirm whether this potential of the ICM can be realised at all and, if so, under what conditions. The use of virtual patients, case-based teaching and learning methods and communication training could be integrated into the ICM to this end [7]. In the opinion of the authors, the reasons for the success measured in the (predominantly non-medical) studies undertaken to date and the high acceptance levels of the Inverted Classroom Model are rooted in the following aspects:

- It is learner-centred and facilitates accelerated and independent learning.
- It incorporates technical innovations (screencasts).
- The Open Educational Resources movement fits nicely into the ICM and can be made an integral component of the method. It allows for adaptation to the learning behaviour of the learner.
- It offers spaces for discussion and knowledge assimilation.

Not least, the ICM differs from many blended learning scenarios in that it gives concrete indications concerning configuration of learning phases. Instructors using these methods must, to a greater extent than usual, work out a didactic concept prior to the course in which the principle of congruence between learning objectives and teaching methods as well implementation can actually be executed. In this context, there is considerable leeway for instructors and learners. The positive results, studies and projects related to learning performance as well as the high acceptance level outlined above should serve as an incentive to test and apply this new form of teaching. The projects that have been undertaken to date can serve as an example of good practice for education and training in health professions. Particularly experts in education research are called for to, by means of methodically rigorous studies, confirm the actual benefit and to more closely specify the necessary parameters for an effective implementation. Corresponding studies in which student learning performance represents the principal outcome parameter can provide a basis for practical recommendations. Concluding this introduction to the ICM and despite the absence of such reliable data, the authors would like to offer interested readers several tips that may be helpful when implementing ICM instruction. The following tips are based on information from publications of the Vanderbilt Center for Teaching, Bram and the University of Southern California [http://cft.vanderbilt.edu/guides-sub-pages/flipping-the-classroom/ cited 27. Mai 2014].

1. The students must be provided the opportunity to acquire factual knowledge prior to the classroom phase. Short educational videos and accessing existing instructional material in terms of Open Educational Resources are helpful, provided they are matched to the respective learning objectives of the specific course. The use of other, non-digital material is, however, equally possible. The course concept must make the thematic connection between online and classroom phase clear. Only then can the students recognise and utilise the advantages of the method.
2. Incentive systems should be created to stimulate students to address the contents before the classroom phase. For example, active forum discussions with students or quizzes could be given marks or assessed.
3. Assessment instruments must be implemented in the process in order to give feedback to the students on their knowledge and learning performance and to allow instructors to get an idea of the knowledge levels of the respective user.
4. Activities in the online phase and classroom phase must be well-structured. Students can deal with the concept more easily when content and time requirements are firmly defined.
5. Instructors should not repeat content from the online phase at the beginning of the classroom phase; they should only respond to questions.
6. Instructors should facilitate and support the creation of learning groups and supervise them.
7. Feedback from instructors and students is essential to the success of the ICM. Feedback on learning progress should be given iteratively during the entire process; this holds for the online phase as well.
8. Implemented technology should be easy-access and ideally already familiar to the users.

The need for the systematic examination from the outset even of recent developments such as the ICM is apparent. Likewise with regard to Gartner’s Hype Cycle, it is urgently necessary to push foundational research in this field in order to prevent it from fading to obscurity because of lack of theoretical grounding [http://www.gartner.com/newsroom/id/2819918 cited 12. Januar 2015](17). The first, quite inhomogeneous research reports in the field show promising results and should urgently be tested and examined for use in medical education and training in other health professions as well. Particularly the synergy between the ICM and accelerated learning as well as clinical reasoning, - so relevant in medical education - should undergo a more in-depth analysis. Furthermore, it is essential to ascertain which learning domains are being addressed and whether perhaps even affective learning objectives can be communicated using the ICM.

## Competing interests

The authors declare that they have no competing interests. 

## References

[1] Fabry G, Fischer MR (2014). Das Medizinstudium in Deutschland–Work in Progress. GMS Z Für Med Ausbild.

[2] Ellaway R, Masters K (2008). AMEE Guide 32: e-Learning in medical education Part 1: Learning, teaching and assessment. Med Teach.

[3] Mehta NB, Hull AL, Young JB, Stoller JK (2013). Just Imagine: New Paradigms for Medical Education. Acad Med.

[4] McLaughlin JE, Roth MT, Glatt DM, Gharkholonarehe N, Davidson CA, Griffin LM, Esserman DA, Mumper RJ (2014). The flipped classroom: a course redesign to foster learning and engagement in a health professions school. Acad Med.

[5] Prober CG, Heath C (2012). Lecture halls without lectures--a proposal for medical education. N Engl J Med.

[6] Prober CG, Khan S (2013). Medical Education Reimagined: A Call to Action. Acad Med.

[7] Van der Vleuten CP, Driessen EW (2014). What would happen to education if we take education evidence seriously?. Perspect Med Educ.

[8] Wissenschaftsrat (2014). Empfehlungen zur Weiterentwicklung des Medizinstudiums in Deutschland auf Grundlage einer Bestandsaufnahme der humanmedizinischen Modellstudiengänge.

[9] Arum R, Roksa J (2011). Academically Adrift: Limited Learning on College Campuses.

[10] Berwick DM, Finkelstein JA (2010). Preparing Medical Students for the Continual Improvement of Health and Health Care: Abraham Flexner and the New "Public Interest". Acad Med.

[11] Irby DM, Cooke M, O'Brien BC (2010). Calls for Reform of Medical Education by the Carnegie Foundation for the Advancement of Teaching: 1910 and 2010. Acad Med.

[12] Speedie MK, Baldwin JN, Carter RA, Raehl CL, Yanchick VA, Maine LL (2012). Cultivating 'habits of mind' in the scholarly pharmacy clinician: report of the 2011-12 Argus Commission. Am J Pharm Educ.

[13] Becker WE, Watts M, Becker SR (2006). Teaching Economics: More Alternatives to Chalk and Talk.

[14] van Treeck T, Himpsl-Gutermann K, Robes J, Ebner M, Schön S (2013). Offene und partizipative Lernkonzepte. E-Portfolios, MOOCs und Flipped Classrooms. L3T Lehrbuch für Lernen und Lehren mit Technologien.

[15] Lorenz A, Einert A, Dinter B, Kawalek J, Hering K, Schuster E (2012). FC WInf: Flipped Classroom in der Wirtschaftsinformatik. Wissenschaftliche Berichte 114 - 2012, Band 2582–2599..

[16] European Commission (2014). Report to the European Commission on New modes of learning and teaching in higher education.

[17] Bergmann J, Sams A (2012). Flip Your Classroom: Reach Every Student in Every Class Every Day.

[18] Lage MJ, Platt GJ, Treglia M (2000). Inverting the classroom: A gateway to creating an inclusive learning environment. J Econ Educ.

[19] Handke J (2012). Das Inverted Classroom Model: Begleitband zur ersten deutschen ICM-Konferenz.

[20] Johnson L, Adams Becker S, Estrada V, Freeman A (2014). NMC Horizon Report: 2014 Higher Education Edition.

[21] Anderson LW, Krathwohl DR (2001). A taxonomy for learning, teaching, and assessing: A revision of Bloom's taxonomy of educational objectives, abridged edition.

[22] Means B, Toyama Y, Murphy R, Bakia M, Jones K (2009). Evaluation of Evidence-Based Practices in Online Learning: A Meta-Analysis and Review of Online Learning Studies.

[23] Schreiber BE, Fukuta J, Gordon F (2010). Live lecture versus video podcast in undergraduate medical education: A randomised controlled trial. BMC Med Educ.

[24] Prunuske AJ, Batzli J, Howell E, Miller S (2012). Using Online Lectures to Make Time for Active Learning. Genetics.

[25] Nast A, Schäfer-Hesterberg G, Zielke H, Sterry W, Rzany B (2009). Online lectures for students in dermatology: A replacement for traditional teaching or a valuable addition?. J Eur Acad Dermatol Venereol.

[26] Burnette K, Ramundo M, Stevenson M, Beeson MS (2009). Evaluation of a web-based asynchronous pediatric emergency medicine learning tool for residents and medical students. Acad Emerg Med.

[27] Freeman S, O'Connor E, Parks JW, Cunningham M, Hurley D, Haak D (2007). Prescribed active learning increases performance in introductory biology. CBE Life Sci Educ.

[28] Bonwell CC, Eison JA (1991). Active Learning: Creating Excitement in the Classroom.

[29] Bransford JD, Brown AL, Cocking RR (1999). How people learn: Brain, mind, experience, and school.

[30] O'Dowd DK, Aguilar-Roca N (2009). Garage demos: using physical models to illustrate dynamic aspects of microscopic biological processes. CBE Life Sci Educ.

[31] Walvoord BE, Anderson VJ (2009). Effective Grading: A Tool for Learning and Assessment in College.

[32] Handke J, Schäfer AM (2012). E-Learning, E-Teaching und E-Assessment in der Hochschullehre: Eine Anleitung.

[33] Carlisle MC (2010). Using You Tube to Enhance Student Class Preparation in an Introductory Java Course.

[34] Day JA, Foley JD (2006). Evaluating a Web Lecture Intervention in a Human ndash;Computer Interaction Course. IEEE Trans Educ.

[35] Gannod GC, Burge JE, Helmick MT (2008). Using the inverted classroom to teach software engineering.

[36] Fischer M, Spannagel C, Desel J, Haake JM, Spannagel C (2012). Lernen mit Vorlesungsvideos in der umgekehrten Mathematikvorlesung. DeLFI 2012 – Die 10 E-Learning Fachtagung Informatik der Gesellschaft für Informatik e V..

[37] Bruff DO, Fisher DH, McEven KE, Smith BE (2013). Wrapping a MOOC: Student Perceptions of an Experiment in Blended Learning. MERLOT JOLT.

[38] Kerres M (2012). Mediendidaktik: Konzeption und Entwicklung mediengestützter Lernangebote.

[39] Lindner M (2013). Wie macht man MOOC-Videos im Khan-Style?.

[40] Handke J, Franke P, Schulmeister R (2013). xMOOCs im Virtual Linguistics Campus. MOOCs-Massive Open Online Courses: Offene Bildung oder Geschäftsmodell?.

[41] Kopp M, Ebner M, Nagler W, Lackner E, Ebner M, Schön S (2013). Technologie in der Hochschullehre. Rahmenbedingungen, Strukturen und Modelle. Lehrbuch für Lernen und Lehren mit Technologien.

[42] Guo PJ, Kim J, Rubin R (2014). How video production affects student engagement: An empirical study of mooc videos. Proceedings of the first ACM conference on Learning@ scale conference [Internet].

[43] Lucius K, Spannagel J, Spannagel C, Rummler K (2014). Hörsaalspiele im Flipped Classroom. Lernräume gestalten – Bildungskontexte vielfältig denken.

[44] Bishop JL, Verleger MA (2013). The flipped classroom: A survey of the research. https://www.asee.org/public/conferences/20/papers/6219/view.

[45] Raupach T, Grefe C, Brown J, Meyer K, Schuelper N, Anders S (2015). Moving Knowledge Acquisition From the Lecture Hall to the Student Home: A Prospective Intervention Study. J Med Internet Res.

[46] Deslauriers L, Schelew E, Wieman C (2011). Improved Learning in a Large-Enrollment Physics Class. Science.

[47] Hartley J, Cameron A (1967). Some Observations on the Efficiency of Lecturing. Educ Rev.

[48] Kononowicz AA, Hege I, Safeeullah S (2010). Virtual patients as a practical realisation of the e-learning idea in medicine. E-learning, experience and future.

[49] Morgan H, McLean K, Chapman C, Fitzgerald J, Yousuf A, Hammoud M (2015). The flipped classroom for medical students. Clin Teach.

[50] Heiman HL, Uchida T, Adams C, Butter J, Cohen E, Persell SD, Pribaz P, MacGaghie WC, Martin GJ (2012). E-learning and deliberate practice for oral case presentation skills: A randomized trial. Med Teach.

[51] Tune JD, Sturek M, Basile DP (2013). Flipped classroom model improves graduate student performance in cardiovascular, respiratory, and renal physiology. Adv Physiol Educ.

[52] Street SE, Gilliland KO, McNeil C, Royal K (2014). The Flipped Classroom Improved Medical Student Performance and Satisfaction in a Pre-clinical Physiology Course. Med Sci Educ.

[53] Tolks D, Pelczar I, Bauer D, Brendel T, Görlitz A, Küfner J, Simensohn A, Hege I (2014). Implementation of a Blended-Learning Course as Part of Faculty Development. Creat Educ.

[54] Gerhardt-Szep S (2013). Praxisbeispiel P@L?: Erprobung eines neuen Lernformates.

[55] March PL, McPherson A (1996). The important attributes of a nurse from the perspective of qualified and student nurses. J Adv Nurs.

[56] Thomas EJ, Sexton JB, Helmreich RL (2003). Discrepant attitudes about teamwork among critical care nurses and physicians. Crit Care Med.

[57] Shin K, Jung DY, Shin S, Kim MS (2006). Critical thinking dispositions and skills of senior nursing students in associate, baccalaureate, and RN-to-BSN programs. J Nurs Educ.

[58] Middleton-Green L, Ashelford S (2013). Using Team-Based Learning in Teaching Undergraduate Pathophysiology for Nurses. Health Soc Care Educ.

[59] Lake DA (2001). Student Performance and Perceptions of a Lecture-based Course Compared With the Same Course Utilizing Group Discussion. Phys Ther.

[60] Pierce R, Fox J (2012). Vodcasts and Active-Learning Exercises in a Flipped Classroom" Model of a Renal Pharmacotherapy Module. Am J Pharm Educ.

[61] Persky AM, Dupuis RE (2014). An Eight-year Retrospective Study in "Flipped" Pharmacokinetics Courses. Am J Pharm Educ.

